# Producer Perceptions of the Prevention of Tail Biting on UK Farms: Association to Bedding Use and Tail Removal Proportion

**DOI:** 10.3390/ani9090628

**Published:** 2019-08-29

**Authors:** Anna Valros, Claire Barber

**Affiliations:** 1 Research Centre for Animal Welfare & Department of Production Animal Medicine, Faculty of Veterinary Medicine, University of Helsinki, P.O. Box 57, 00014 Helsinki, Finland; 2 Animal Science (Health & Welfare), Agricultural and Horticultural Development Board, Stoneleigh Park, Kenilworth CV8 2TL, UK

**Keywords:** pig, tail biting, tail docking, farmer perceptions, risk factors

## Abstract

**Simple Summary:**

Tail biting is a serious behavioural problem in modern pig production which causes both animal welfare challenges and economic losses. The aim of this study was to collect information on the perceptions of farmers on how to best prevent tail biting, and on their attitudes towards tail biting and docking. Further, the aim was to investigate if perceptions are influenced by the specific system of farming, with a focus on different levels of bedding use and different tail docking practices. To achieve the goal, pig producers in the UK were surveyed. The results show that producers rank the importance of preventive measures differently to scientists and other experts. This calls for consideration when communicating with producers, and for further consideration of producer knowledge, which might be based on a more holistic view than that of researchers. The study also shows that the perception of how to best prevent and intervene to avoid tail biting differs between farms of different types, and that these perceptions might be influenced by the farmers’ own experiences, which again, might differ between countries and farming systems.

**Abstract:**

Tail biting causes widespread problems both for animal welfare and in the form of economic losses in pig production. This study was performed to better understand the perceptions of farmers on how to best prevent tail biting, and if perceptions are influenced by the specific system of farming, with a focus on different levels of bedding use and docking different proportions of the tail of their pigs. Pig producers in the UK were surveyed on their perceptions of the efficacy of preventive measures and attitudes towards tail biting and docking. In total, 204 responses were included. The results show that producers rank the importance of preventive measures differently to scientists and other experts. This calls for consideration when communicating with producers; and for better integration of knowledge based on practical experiences with scientific results. The study also shows that the perception of how to best avoid tail biting differs between farms of different types, and that these perceptions might be influenced by the farmers´ own experiences—one example being that farms currently using plentiful amounts of bedding also value this more highly as a way to avoid tail biting than those that do not.

## 1. Introduction

Tail biting is a serious and common behavioural problem in modern pig production. Tail biting damage results in both production losses and reduced animal welfare, and it is therefore important that tail biting is efficiently prevented (for a review, see [1]). In most countries in Europe and beyond, tail biting is prevented by docking the tail of pigs [2]. Tail docking, even though it does reduce the occurrence of tail biting, does not eliminate the problem [3]. Tail docking is further problematic from two points-of-view: Firstly, it is a painful procedure [4,5,6], even though probably less painful than serious tail biting [7]. Secondly, and probably most importantly, tail biting is a multifactorial problem, and the risk factors include challenges in the housing and management of pigs, such as suboptimal climate conditions, problems with feeding and nutrition, health problems (clinical and subclinical) or lack of manipulable material [1]. Tail docking has thus been suggested to reduce the adverse effects of keeping pigs under suboptimal conditions and management [7,8,9]. 

Recently, several activities have been ongoing in the EU to achieve a stricter enforcement of the existing ban on routine tail docking in the EU [2,10]. However, despite these activities, there has been very little actual reduction in the proportion of pigs being docked both in the EU overall, and in the UK [2]. One reason for the difficulty in achieving progress might lie in producers being reluctant to try non-docking [10,11]. Nalon and DeBriyne [10] suggested that this might be due to the multifactorial nature of tail biting, making it difficult to control and predict. To achieve behavioural change, the motivation of the producer, as well as knowledge about how to prevent tail biting and deal with outbreaks, are essential [12].

It has been shown that scientists and farmers disagree somewhat regarding how they rank risk factors for tail biting [11,13]. Suggested reasons for this discrepancy are that farmers may have a more holistic view of the problem, or that farmers might partly ignore scientific information because it is not concrete enough, or focuses on specific factors only [14]. To increase the understanding of how to achieve behavioural change in order to reduce the need for tail docking, it is therefore important to also understand how farmers perceive the problem, and which preventive methods they find most important on their farms. There is, however, only limited information available on this topic, and perceptions might differ between countries, due to differences in factors such as societal attitudes, traditions and practices. Previous studies on farmer perceptions in relation to tail biting and docking from the Netherlands [11], Finland [13] and Sweden [15] indeed indicate some differences between countries. 

This study is based on the methodology used in Valros et al. [13], where Finnish producers were surveyed for their ranking of different tail biting preventive measures and their attitudes towards tail biting and docking. In Finland, tail docking has been banned since 2003, which means the study included farms rearing undocked pigs only. Views of farmers might be influenced by the system farmers are used to [16], as well as by the previously mentioned differences between countries. The aim of the current study was to increase the understanding of whether producers used to different productions systems, and with differing experiences of docking tails, perceive the effectiveness of preventive measures for tail biting differently. Further, the aim was to assess whether these factors are associated with differing attitudes towards tail biting and docking. The UK provides an interesting study population, as there is a great variation in both docking practices and production systems within the country.

## 2. Material and Methods

The questionnaire was slightly modified to fit UK conditions from that used in Valros et al. [13], which was designed to be as short, and as easy as possible for the producers to fill in. The original questionnaire is included as Appendix A. It included a few general questions (farm type; number of finisher places; farmer experience; number of pigs in a typical pen; and ratio of stock-keepers to pigs). The questionnaire then included sets of questions to evaluate the perceived importance of preventive measures and intervention measures (defined as measures taken when tail biting occurs in a pen), using a scale from 1 (not important) to 7 (extremely important)). The preventive measures were divided into four subcategories: ***Housing and environment; Pig behaviour; Feed and water; Animals.*** Furthermore, farmers were asked to evaluate the efficacy of different manipulable materials and objects in preventing tail biting on the scale 1 (not effective) to 7 (extremely effective). In the questionnaire, these were grouped as (a) ***Bedding and chewable materials***, and (b) ***Objects***. In the last part of the questionnaire, producers were asked how serious a problem they perceive tail biting to be on their farm (1—not a serious problem, to 7—an extremely serious problem) and how much tail biting they perceive to be manageable in their unit, using preset categories ranging from 0% to over 15% (see Appendix A). Respondents were also asked to provide information about the level of tail biting in their most recent batch, and to indicate the proportion of tail removal on their farm (from none to >½ of the tail removed). Tail biting was defined as “a tail with clear biting marks and/or blood, or the tail is clearly shortened due to biting”. Respondents were further given the opportunity to write open comments and suggestions on the above topics. 

The questionnaire was available for completion both as a paper version and online in Survey Monkey. A hardcopy of the questionnaire and an explaining cover letter was sent out by post to all 1792 UK pig farms, which had registered pig movements on the eAML2 system (the electronic pig movement reporting service which is a requirement in England) in the six months prior to the survey beginning. Thus, the questionnaire was open to the majority of active pig producers, including farms of varying size and both indoor and outdoor systems. To encourage responses a pre-paid response envelope was included. The Agriculture and Horticulture Development Board (AHDB) promoted the survey on the AHDB Pork website and through an e-newsletter sent to pig producers. All paper responses were entered on to Survey Monkey to collate all responses in one file. The survey was open to responses for a six months period (15 November 2016–15 May 2017). No reminders were sent out, but information on the study was repeatedly included in the weekly AHDB Pork e-newsletter *Pork News.*

### Statistical Analyses

All statistical analyses were performed with IBM SPSS Statistics for Windows, Version 21.0. (IBM Corp., Armonk, NY, USA.)

All statistical tests were performed using non-parametric tests, as most variables were non-normally distributed, and as the majority of the variables are non-continuous. However, descriptive data on the scoring of importance of the different preventive and intervention measures as well as of effectiveness of different manipulable materials are given both as median and interquartile range and as mean and standard deviation. Further, because the median is rather invariable, and thus does not discriminate differences very well in data based on a categorical scale, these measures are ranked using mean values. Overall means for the four subcategories of preventive measures were compared using Friedman’s Two way analyses, followed by the Wilcoxon signed rank sum test for pairwise comparisons when appropriate. 

Differences in the perceived importance of different prevention and intervention measures, as well as of perceived effectiveness of manipulable materials between farms using different amounts of bedding and farms rearing pigs with differing proportion of tail removal were tested using independent sample Kruskall—Wallis-tests followed by Bonferroni-corrected pairwise comparisons, when appropriate. For prevention measures, both overall means of subcategories and separate measures were tested.

A Kruskall–Wallis test was also used to assess differences in perceived seriousness of the problem of tail biting between farms with differing proportion of tail removal. Associations between the level of tail biting thought to be manageable, occurrence of tail biting on the farm, and proportion of the tail that is docked were tested with Spearman rank correlations, as was the effect of unit size and ratio of stock-keepers to pigs with these variables. 

Significance level was set at *p* < 0.05, and only significant results are presented.

Open comments were analyzed manually by grouping comments of similar nature. For example, all suggestions for adding salt as an intervention when tail biting outbreaks occur were considered as one “group”, irrespective of the form of salt addition suggested, and comments underlining the importance of tail docking in any way were grouped. The number of comments in each “group” are given, as well as the total number of comments that had been given for that section of the questionnaire. In addition, for some topics, a few representative comments were chosen as example quotes to further illustrate how producers worded their opinions.

## 3. Results 

In total, the results included 204 responses, the response rate being 11.4%. The responses represent 72 finishing farms, 41 rearing/finishing farms, 89 farrow to finish farms and 9 breeding farms. Some respondents had indicated more than one option for farm type, thus the sum is not the same as total number of farms. Of the farms, 178 kept their finishing pigs indoors. Finishing pigs were kept outdoors on 10 farms (defined as *Pigs are born outside in fields, and stay outside in open air pens. Space is more restricted than in free range systems*) and 8 had free range pigs (defined as *Pigs are born outside, remain outside, and are free to roam*). In addition, 4 farms had a combination of these, and 6 farms indicated they did not have finishing pigs at all. Outdoor farms in the current sample were considered too few to allow for meaningful statistical comparisons between indoor and outdoor farms. 

The number of finishing pig places on the farm varied between 0 and 20,000 places, with a mean of 2161 pig places. Some respondents had pigs in several units. If the information on number of finishing pig places was given as a sum for several units, farm size was, however, set as a missing value. The distribution of farm sizes is shown in Table 1. 

The respondents had on average 28 years of experience of pig production (range 1–63 years). On average the ratio of caretakers to pigs was 1:633 (range 1:25–1:4000). 

On average farms had 47.6 pigs per pen, with a range of 4.5–300 pigs per pen. For farms with finisher pigs only, the mean number of finisher pigs per pen was 50.4 (4.5–300). If farms indicated several pen sizes, close to each other, the mean was used, and if the pens varied greatly—e.g., 20 to 400 pigs per pen, the value was set as missing. 

The distribution of the level of bedding the farms used, as well as the distribution of farms with differing proportion of tail removal is shown in Table 2 and Table 3. There is an interaction between the proportion of tails docked and bedding amount used, with none of the farms rearing pigs with intact tails using no bedding, while 57% of farms docking >½ of the tail reported no use of bedding. The corresponding figures for using deep-litter systems was 65% and 36%, for no docking and docking >½ of the tail, respectively. 

Only about half of the respondents had chosen to indicate the level of tail biting in their last batch. Of these, some had given non-numerical replies, or replies that could otherwise not be translated into a reliable % per batch. These values were set as missing. Thus, only 103 of the 204 responses giving an indication of tail biting incidence in their last batch were used in the analyses. Further, a few respondents had only replied that they had below 1% of tail biting lesions. For analyses, this was set as 0.5% for all. On average 0.7% of the pigs in the previous batch had bitten tails (range 0–22%). 

### 3.1. Perceived Importance of Prevention Measures 

Descriptive data on the perceived importance of the different preventive measures is reported in Table 4. To allow for a comparison to the similar data collected in Finland [13]) a column with rankings of the same measures by Finnish farmers has been included. Only 3 of the 20 included preventive measures got mean scores above 6, while 12 of the measures scored over 5. The overall mean score differed between subcategories (Friedman’s two-way analysis *Q* (3) = 66, *n* = 167, *p* < 0.001). This was explained by the subcategory ***Housing and environment*** scoring significantly lower (median 4:9, interquartile range: 1.7) than that for any of the other subcategories (*p* < 0.001 for all pairwise comparisons). The median subcategory mean score for ***Pigbehaviour*** was 5.4 (1.6), for ***Feed and water*** 5.5 (2.5) and for ***Animals*** 5.8 (1.8). 

**Figure 1:**
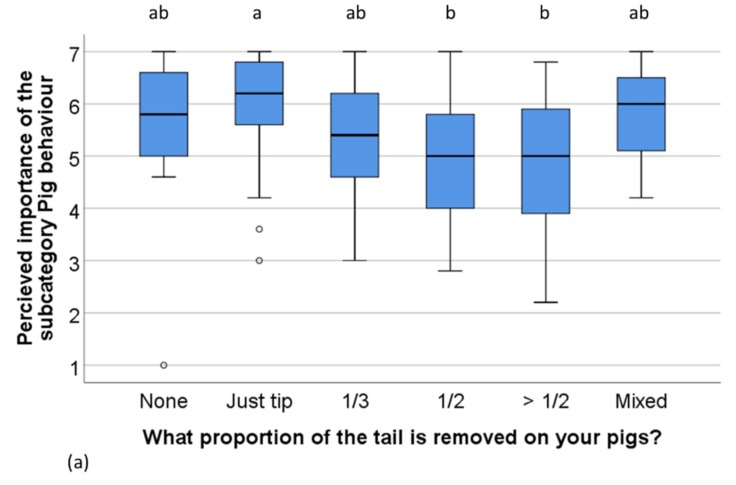

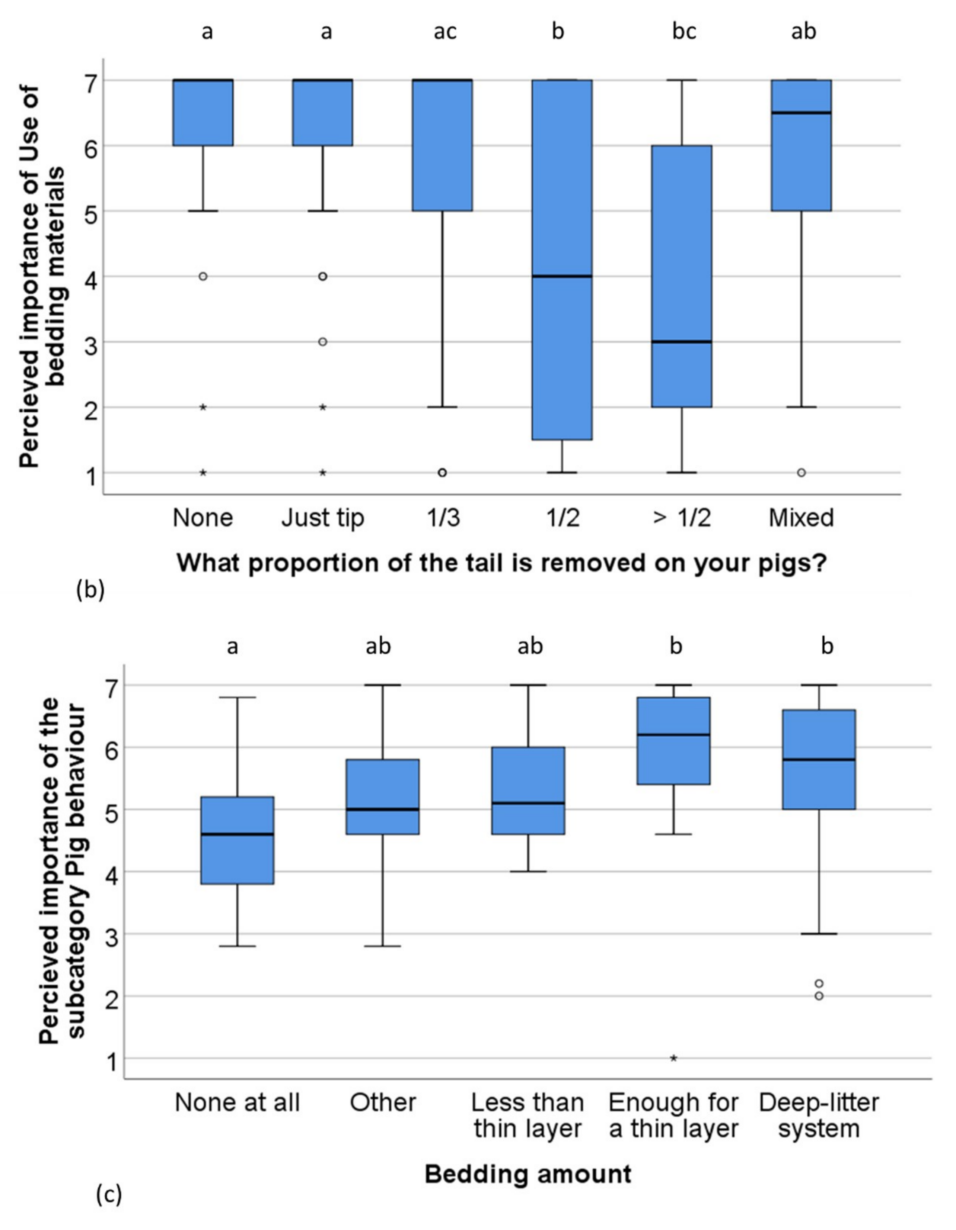
Distribution of overall mean rating of factors within the ***Pig behaviour*** subcategory (**a**) and the rating of the measure *Use of bedding* (**b**) of respondents (*n* = 176) which rear pigs with different proportions of the tails removed. (**c**) shows the distribution of overall mean rating of factors within the ***Pig behaviour*** subcategory of respondents using different amounts of bedding on their farms (*n* = 181). Scale: 1 (not important) to 7 (extremely important). The boxplot indicates medians and quartiles (25% and 50%) as well as outliers. Boxes lacking common letters differ (*p* < 0.05).

The overall mean scoring of the subcategory ***Pig behaviour*** was related to the proportion of tail that was docked on the farm (Kruskall–Wallis *x*^2^ (5) = 20, *n* = 176, *p* = 0.001) (Figure 1a). This difference appeared to be mainly explained by different scores in the factor *Use of bedding* (Kruskall–Wallis *x*^2^ (5) = 24, *n* = 155, *p* < 0.001) between farms with different docking length, as this was the only separate measure that showed a difference (Figure 1b). Further, the overall mean rating of ***Pig behaviour*** was also related to the amount of bedding used on the farm (Kruskall–Wallis *x*^2^ (4) = 35, *n* = 181, *p* < 0.001). Farms using enough bedding material to just cover the whole pen when thinly spread and farms with deep litter rated this subcategory highest (Figure 1c). The overall ratings of the other subcategories did not differ between farms with different docking lengths, or farms using different amounts of bedding. 

The respondents were asked to suggest further factors which contribute to preventing tail biting in their unit as open answers. In total 108 respondents commented, and of these 25 responded that they had no further suggestions. Tail docking was the topic most commonly occurring in the comments: 17 respondents mentioned tail docking as such, and 7 respondents suggested the importance of tails being an even length. Factors related to enriching the environment, including outdoor access, adding soil, bedding, cardboard boxes or toys, having radio or music on, or frequently changing materials were mentioned in 16 answers. Other issues that were mentioned less frequently included, among others, breed and genetics (6), health issues, including vaccinations (6), adding salt in feed or as blocks (5), low level of disturbance and stress, including avoiding staff changes (5), and grouping strategies of pigs, including separating small or bitten pigs and separating sexes (5). Changing or difficult weather conditions were mentioned as risk factors by 5 respondents, overstocking by 4 respondents and poor quality of pigs or the occasional “abnormal pig” by 4 respondents. In addition, climate issues, such as temperature, humidity, and ventilation were mentioned more sporadically. 

### 3.2. Perceived Importance of Intervention Measures

The distribution of ratings for the different intervention measures are shown in Table 5. *Identifying the biter*, *Removing the biter from the pen* and *Removing the bitten pig(s) from the pen* were all scored on average 6 or above. Within all these three interventions, there was a high level of agreement between the respondents. *Adding objects for manipulation* and *Adding bedding material* were also rated as rather important (over 5), but the scoring appeared to divide respondents more, especially for *Adding bedding material.* The other intervention measures all scored below 5 with *Use of anti-biting substances on the tail* getting the lowest mean score. The respondents were further asked to specify which anti-biting substance they use. In total, 83 respondents had wrtten some comment. Of these, some were irrelevant for the topic, and some just stated that they had no experience of anti-biting substances, leaving 73 usable replies. The most frequently used substance was Tail guard (33 responses), Tar (23) and Porcivet (10). Six respondents wrote in open answers that anti-biting substances are not effective, and one that they might even attract pigs to the tail. 

**Figure 2:**
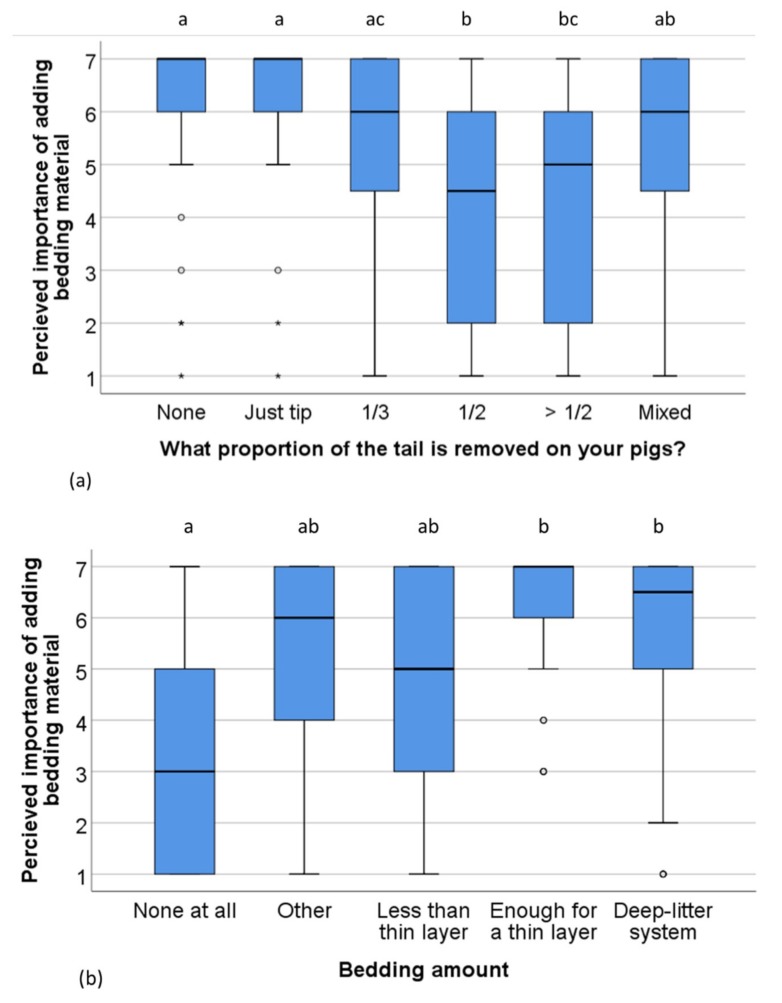
Scoring of the importance of *Adding bedding material* as an intervention measure when tail biting occurs in a pen on (**a**) farms which rear pigs with different proportions of the tails removed (*n* = 176) and (**b**) using different bedding amounts (*n* = 180). Scale: 1 (not important) to 7 (extremely important). The boxplot indicates medians and quartiles (25 and 50%) as well as outliers. Boxes lacking common letters differ (*p* < 0.05).

Farms with different docking lengths scored the measure *Removing the bitten pig(s)* differently (Kruskall–Wallis *x*^2^ (5) = 13, *n* = 175, *p* = 0.02), but there was no clear pattern in the difference, with pairwise comparisons only revealing a difference between farms with mixed docking lengths and farms docking only the tip (*p* = 0.045). Also *Adding bedding material* was scored differently by farms with different docking length (Kruskall–Wallis *x*^2^ (5) = 29, *n* = 176, *p* < 0.001). Farms that had intact- tailed pigs, or only docked the tip of the tail scored this as more effective than farms that docked a larger proportion of the tail (Figure 2a). *Use of anti-biting substances on the tail* was also rated differently by farms with different docking lengths (Kruskall–Wallis *x*^2^ (5) = 17, *n* = 151, *p* = 0.004). Numerically, farms with intact tails rated this measure lower than farms with docked tails, but the only significant pairwise comparison was between farms docking none and farms docking 1/3 of the tails (*p* = 0.002). Farms using different levels of bedding scored the *Adding bedding material*-measure differently (Kruskall–Wallis *x*^2^ (4) = 45, *n* = 180, *p* < 0.001, Figure 2b). There was no difference between farms with different level of bedding materials and docking length in any of the other intervention measures.

In total, 58 of 90 comments included suggestions on additional intervention measures, while 32 respondents commented that they had no further suggestions. Other intervention measures suggested by the respondents in open answers included adding salt to feed, or as licks (16 responses), checking and adjusting the “basics” such as ventilation, feed and water availability, and stocking density (7), adding more manipulable materials or objects, such as soil, fodder beets, extra straw, meal on the floor, paper bags, fresh tree branches, plastic cans with stones inside (‘rattles’), or chains (14). Seven respondents mentioned different measures related to animal grouping as being effective, including segregation of pigs, moving pigs to a new pen, keeping pigs with pigs they know, separating pigs by sex and splitting the group to identify the biter (7). A few (3) respondents also mentioned the importance of increased vigilance by the caretaker.

### 3.3. Perceived Effectiveness of Different Manipulable Materials for Preventing Tail Biting

The results on scorings of perceived effectiveness of manipulable materials are given in Table 6. Of all the manipulable materials, only *Straw* scored on average over 5. *Cardboard or paper sacks* was the only other ***Bedding and chewable material*** scoring above 4, while most ***Objects*** scored between 4 and 5. 

**Figure 3:**
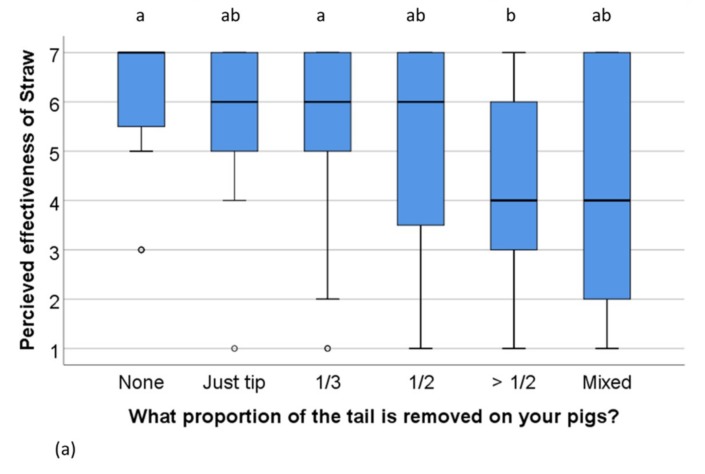

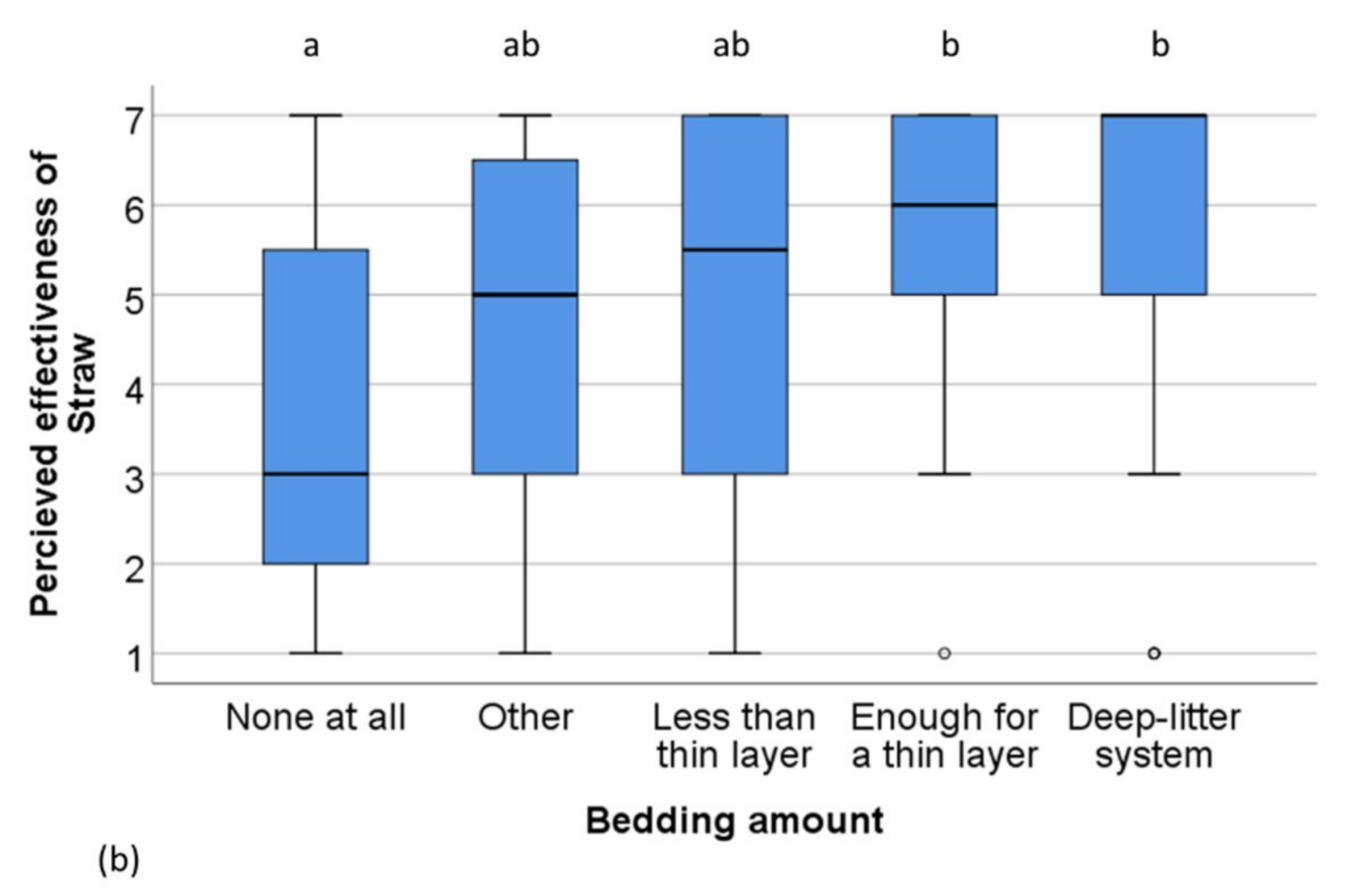
Scoring of the effectiveness of *Straw* as a manipulable material to prevent tail biting on (**a**) farms which rear pigs with different proportions of the tails removed (*n* = 155) and (**b**) farms using different bedding amounts (*n* = 162). Scale: 1 (not effective) to 7 (extremely effective). The boxplot indicates medians and quartiles (25% and 50%) as well as outliers. Boxes lacking common letters differ (*p* < 0.05).

The perceived effectiveness of *Straw* differed between farms with different docking length (Kruskall–Wallis *x*^2^ (5) = 24, *n* = 155, *p* < 0.001, Figure 3a). The perceived effectiveness of *Straw* also differed between farms using different amounts of bedding: The more bedding was used, the higher the perceived effectiveness of *Straw* (Kruskall–Wallis *x*^2^ (4) = 42, *n* = 162, *p* < 0.001, Figure 3b). The effectiveness of using *Balls or other objects loose on the floor* was also perceived differently by farmers using different amounts of bedding (Kruskall–Wallis *x*^2^ (4) = 14, *n* = 135, *p* = 0.008), but the only difference in pairwise comparisons was between farms using other types of bedding and farms using deep litter (*p* = 0.01). There was no difference between farms with different level of bedding materials and docking length in the perceived effectiveness of any of the other manipulable materials.

The respondents also asked to specify any other materials they had used on their farm to prevent tail biting. A total of 80 comments were given, but 27 of these were just to say they had not further suggestions. The most frequently mentioned option was adding soil or turf (13) to the pen, followed by salt rocks or pig licks (12) and empty plastic containers of different types, either used as such (12), or filled with stones or other objects to make a rattling sound (4) or water (1). Also, plastic (8) and hose pipes (2) were rather popular amongst respondents. Other materials or objects that got more than one mention in the open answers included feed sacks (5), wellington boots (4), tyres (4), vegetables, such as fodder beet and turnips (4) and tree branches or logs (3), straw (3) and newspaper (2). 

### 3.4. Producer Attitudes towards Tail Biting

Of the respondents, 30% had chosen the option “0%” and 57% the option “1–2%” for the question “How much tail biting do you perceive as manageable on your unit?”. The perceived level of manageable tail biting was rather evenly distributed over respondents rearing pigs with differing tail lengths, indicating no clear connection between these two variables.

**Figure 4:**
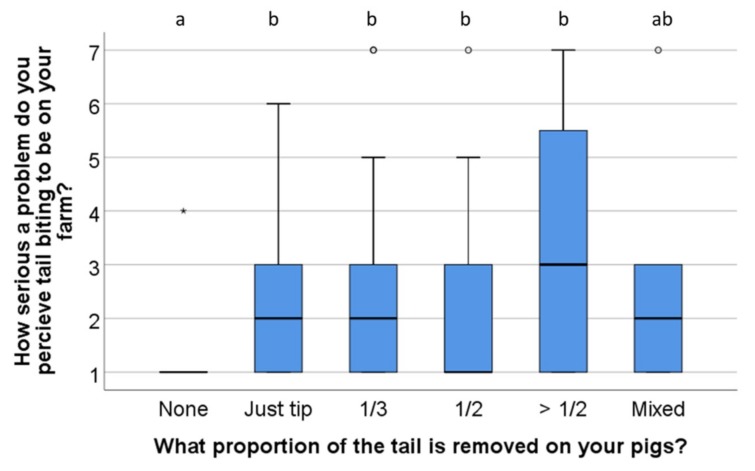
Distribution of scores for perceived seriousness of tail biting problem on the respondents’ farms (*n* = 176), divided by which proportion of tails is docked on the farm (1—not a serious problem to 7—an extremely serious problem). The boxplot indicates medians and quartiles (25% and 50%) as well as outliers. Boxes lacking common letters differ (*p* < 0.05).

The median score for how serious a problem respondents perceived tail biting to be on their farm was 2 (interquartile range: 2, range 1–7) on the scale 1—Not a serious problem to 7—an extremely serious problem. Of the respondents, 57% scored the problem to not be serious (1–2), while 6.9% scored it to be very serious (6–7). The proportion of tail which was docked on the respondent’s farm was related to how serious a problem tail biting was perceived to be (Kruskall–Wallis *x*^2^ (5) = 34, *n* = 176, *p* = 0.001, Figure 4). The respondents that reared long-tailed pigs scored tail biting as a less serious problem than most other docking lengths, while respondents which docked more than ½ of the tail scored numerically highest. 

The reported tail biting incidence in the last batch of the respondents’ unit correlated positively with how serious a problem the respondent thought tail biting was (*r_s_* = 0.36, *n* = 99, *p* < 0.001). In addition, reported tail biting incidence correlated negatively with the ratio of staff per finishing pigs (*r_s_* = −0.27, *n* = 92, *p* = 0.009), meaning that tail biting occurred more on farms with less staff per animal. Even though farm size was negatively correlated with ratio of staff per animals (*r_s_* = −0.62, *n* = 155, *p* < 0.001), with less staff per animal on big farms, farm size per se did not correlate with tail biting incidence (*p* > 0.1). 

For farms which docked differing proportions of the tail of pigs as a continuous variable (excluding farms with mixed tail lengths), there was a positive correlation between farm size class and the proportion of tail docked (*r_s_* = 0.20, *n* = 158, *p* = 0.01), with larger farms having pigs with a larger proportion of their tails docked. The same was also seen for ratio of farm staff per animals (*r_s_* = −0.16, *n* = 144, *p* = 0.05), meaning that a larger part of the tail had been docked on farms with fewer staff per animals. 

Farms that did not dock tails at all reported a much lower prevalence of tail biting in their last batch (mean 0.007%, *n* = 14) than in farms with pigs with any proportion of their tails being docked (overall mean 0.38%, range 0.36–1.3%, *n* = 87). 

### 3.5. General Open Comments

At the end of the questionnaire, respondents were asked for further comments. In total, 93 respondents gave some comment, but 18 of these only indicated that they had no further comments. The contents of the remaining 75 comments varied a lot, but many wanted to underline that tail docking is important, or even essential (18 responses), either for production, to reduce carcass condemnations at slaughter, or, most commonly, for the welfare of the pigs. All of these responses were given by producers that currently raise pigs with docked tails of different lengths. Several respondents (7) also commented that tail biting, or the banning of tail docking is cruel, and not docking is “asking for trouble”. Again, all these responses were from respondents that currently raise pigs with docked tails. Tail docking was not always seen as a simple decision, but based on a weighing of costs and benefits:

‘We don´t like tipping tails, but when we have had a spell of tail biting it is so cruel we feel tipping is better’.
‘Tail biting has far worse welfare outcomes than tail docking’.

Several respondents compared tail docking to vaccination:

‘I haven’t always tail docked and I think not tail docking is the welfare issue. It is like failing to vaccinate’.

Four respondents mentioned that they had tried to raise pigs with long tails at some point, but had gone back to docking due to lots of problems with biting, pigs suffering and high levels of carcass condemnations at slaughter.

In total, five respondents commented that tail biting is not an issue on their farm. Of these, three reported that their pigs have half of their tails docked, while two reared intact-tailed pigs. In addition, seven respondents commented that they have no problems with tail biting either because they have worked a lot to reduce risk factors related to e.g., health, temperature or feed, or because they keep pigs outdoors. Of these, three reared pigs with intact tails. Three respondents commented that according to their experience, tail biting was a bigger problem on deep straw or outside. One respondent seemed to summarize this: *‘Tail biting can occur in all housing types’*, while some indicated a frustration of not being able to control the problems: *‘It is just a mystery to me. No consistent cause’.*

A couple of respondents underlined that the stockperson has the biggest effect on the risk for tail biting, and several respondents underlined the importance of docking tails to an even length. 

## 4. Discussion

The current study shows that producers in the UK considered most of the preventive measures listed in the questionnaire as at least somewhat important, with the mean being over 4 on a scale from 1 (not important) to 7 (extremely important) for all but two measures. The rating of different prevention and intervention measures differed somewhat for respondents representing different types of farms, mainly for questions related to use of bedding. UK producers scored preventive measures in a rather similar way to those in Finland [13]: Of the top-ten rated measures, seven were common for the UK and Finnish respondents. The observed differences might be partly explained by differences in production systems between these countries. 

The preventive measures scoring highest (over 6) were *Water available to all pigs; Providing a stocking density which is appropriate for the pen size*, and *Taking care of animal health*. Especially the two first-mentioned also indicate a rather high agreement between respondents regarding the importance of these measures, with the variation (indicated as SD in Table 4) in scores being lower than for the other measures. This result is somewhat interesting, as water provision is not a factor showing up very high on rankings by experts [17]. Good water access in general is, however, considered a relevant risk factor by the HAT-tool, a husbandry advisory tool that was developed in the UK to help identify risk factors for tail biting [18], and fouled drinkers are further included in the AHDB WebHAT tool [19] as one of the risk factors for tail biting in pigs kept on straw. It is possible experts consider that sufficient water supply self-evident, while producers better understand the practical challenges that sometimes relate to ensuring water supply for pigs. 

An appropriate stocking density is often suggested as important for preventing tail biting (see e.g., [2]) However, the scientific evidence for this appears somewhat inconsistent: While some studies do indicate that stocking density might be important [20,21], it is possibly confounded by other factors, such as feeder space [20]. Dutch producers ranked space allowance as the second most important risk factor in the study by Bracke et al. [10], while *Providing an appropriate stocking density* was only ranked 11th by the Finnish producers in the study by Valros et al. [13]. The difference in ranking between the UK and the Finnish data might be due to the difference in group size commonly seen on farms in the two countries. In Finland, finishing pigs are typically reared in pens of less than 20 pigs per pen; whilst in the UK, there is a greater variation, with larger group sizes on average, based on the farms included in this study. Also, major Finnish slaughterhouse companies have already for several years demanded a lower stocking density for finisher pigs than the EU minimum on their contract farms (0.8–0.9 m^2^). Thus, it can possibly be assumed that Finnish pigs experience a more optimal stocking density by default, and that there might be a smaller variation between farms.

*Taking care of animal health* got a high score (6.0) in both the current questionnaire and in the study of Finnish farmers (6.2) [13] and further scored 3.3 of a maximum of 4 by Dutch farmers in the study by Bracke et al. [11]. Animal health is a topic that has received only minor research attention as a factor related to tail biting [8], even though it is an issue of great importance for the industry, and for animal welfare, in general. Interestingly, health was also not one of the most important practices suggested by veterinarians to prevent tail biting in a recent survey [2]. During the last few years there has been an increased interest in understanding the link between (ill-)health and tail biting, e.g., in the EU-project FareWellDock [22] and the COST action CA15134 GroupHouseNet [23]. Some recent studies do imply a mechanistic link between a reduced health level and an increased risk for tail biting [24,25]. Interestingly, there is a difference between Finland (with no tail docking) and the UK regarding pig health status. Finland is free of several important pig diseases, such as porcine reproductive and respiratory syndrome (PRRS) and has a low prevalence of other diseases like swine dysentery [26]. This is not the case for the UK [27].

The subcategory ***Housing and environment*** got the lowest mean score in both the current study, and in that by Valros et al. [13], while Dutch farmers [11] rated stable climate as the most important risk factor. Ensuring a good microclimate is often mentioned by veterinarians as an important measure to reduce tail biting risks [2]. In the current study, however, separate factors related to stable climate specifically, such as *Appropriate temperature in the pen, Reducing/eliminating draughts* and *Maintaining air quality* received high scores, and were also included in the top-10 measures by Finnish farmers [13]. In contrast, factors related to light and noise were scored low, thus resulting in the low mean score of this subcategory. Light is sometimes mentioned as a risk factor for tail biting, but there is no conclusive evidence on its role [17,28]. Thus, the perceived difference between studies might be due to large variation in the measures included within this subcategory in the current study. 

The subcategory ***Feed and water*** was scored significantly higher than all other subcategories in the Finnish study [13], but did not receive equally high relative scorings in the current study. Interestingly, the factor that got the highest score in the Finnish list was *Enough space around feeder*, which was not included at all on the UK top-ten list. Competition for feeder space has been indicated to be an important risk factor in some epidemiological studies [20,29]. Dutch producers [11] did not rate the feeding system as one of the top-ranking measures for tail biting prevention and the expert opinion by EFSA [17] also does not rank feeding as a top risk factor for tail biting. Among veterinarians taking part in the survey reported by [2], feed and water was the third most commonly mentioned set of practices important for reducing tail biting. The discrepancy here might partly be due to differences in feeding systems between countries. For example, most finishing farms in Finland use long troughs and (liquid) meal feeding instead of feeding automats and ad lib feeding [1]), which is common in many other countries. Indeed, in the UK, the vast majority of pigs are fed ad libitum from feed hoppers [30]. This might also explain why *Feeding always at the same time of day* was included as number 10 on the Finnish list, but not on the UK top-ten list. Instead, the importance of ad libitum feeding was mentioned frequently in the UK open answers. 

Interestingly, neither of the top ten lists include use of bedding or objects for manipulation, although *Use of bedding* is rated as rather important in both the UK (5.1) and in Finland (5.5). Among scientists and other experts, this factor is often rated as the most important, and has also received most research attention [2,8]. Dutch producers similarly did not rank boredom of the pigs as a top risk factor for tail biting [11], and further, Dutch producers have also criticized researchers for focusing too much on manipulable materials, while farmers might see their farm in a more holistic way [14]. 

The diversity of farm systems in the current study allows an interesting comparison of the difference in farmer perceptions used to different types of systems. As evident from the farms taking part in this study, UK farms differ in their use (or not) of straw bedding, group sizes, docking lengths, and by having both indoor and outdoor systems. In this study the focus was on comparing farms with differing docking lengths and level of bedding use. As these two factors were linked (all farms with non-docked pigs also used at least some bedding), it is difficult to totally separate these two factors, and they might reflect a certain farm type. In general, farms that removed a smaller amount of the tail, and farms that used a high level of bedding material perceived considering pig behaviour for reducing the tail biting risk and the use of bedding as both a preventive, and an intervention measure as more important. The same connection was found in relation to the effectiveness of *Straw* as a manipulable material in reducing the risk of tail biting. The difference in ratings between farms with different practices regarding docking length and use of bedding might reflect the fact that producers value most the measures they have (positive) experiences of. Further, farm size and ratio of staff per animal also appear to be linked to docking practices: Big farms and farms with lower staff ratio more often reared pigs with a larger proportion of the tail docked. Docking tails might be a way to compensate for reduced possibilities for early detection and intervention on large farms with low staff to animal ratio [7]. Also, in studies including Finnish and Dutch producers, a more favorable attitude towards docking has been reported in farms of larger size [11,13]. 

There are only a few studies on how to efficiently intervene when a tail biting outbreak occurs. Zonderland et al. [31] compared the effect of removing the biter to adding straw, and found both to be equally effective, but the study did not compare to a control treatment with no intervention strategy. Recent studies from Denmark showed that providing straw when the first signs of biting are evident in a pen, or when there is an outbreak [32,33] can efficiently reduce the escalation of the problem, while not totally stopping it. The rating of intervention measures in the current study corresponds with the use of such measures on UK farms in a study by Hunter et al. [29], which found removing the biter was mentioned by 43% and removal of the bitten pigs by 67% of the respondents. Further, 51% of the respondents in the study by Hunter et al. [29] reported adding manipulable objects, 25% adding anti-biting substances and 16% adding straw. The ranking of the intervention measures is also very much in agreement with that of Finnish farmers [13], with the only difference being that Finnish farmers rated *Adding bedding material* as more important than *Removing the bitten pig from the pen*. This might be due to differences in production systems; very few farms in Finland would have deep-litter systems (only one farm within the Finnish study population [13], while 70 of the UK farms had deep-litter systems). Addition of bedding material might be more efficient on farms not already using a large amount of bedding. However, this is not supported by the fact that in the current data it is the farms using no bedding which score the *Adding bedding material*-measure lower than farms using either a thin layer of bedding (enough bedding material to cover the pen when thinly spread) or having deep-litter systems. A more probable explanation for this could be that farms using no bedding in the UK likely operate fully-slatted systems, and that adding bedding material is not feasible on these farms.

Even though *Straw* was ranked as the most effective manipulable material, UK producers appeared to generally find ***Objects*** to be more effective in preventing tail biting than ***Bedding and chewable materials***. Again, this could be due to the fact that on a large proportion of the respondents´ farms using bedding-type materials is not perceived as feasible due to fully-slatted systems. This is slightly different from the perception of Finnish farmers who seemed to prefer most bedding-type materials over objects. Also, the Finnish farmers [13] rated *Straw* as the best bedding-type material for preventing tail biting (5.6), but gave scores over 5 also to several other bedding-type materials, including *Newspaper* (not included in the UK questionnaire), *Hay*, and *Cardboard or paper sacks*. However, in the Finnish questionnaire [13], none of the manipulable ***Objects*** were scored above 5, and *Chain* was given the highest score (4.8). Straw has been reported as a very efficient measure to reduce tail biting [3,28], and as an efficient way to improve pig welfare [34]. Commonly used chains, on the other hand, have generally not been rated as very good enrichment objects either in studies [34,35], or as ranked by experts [36]. Chains are, however, commonly used by farmers in some countries [11,13], and might thus be scored high, as farmers are used to these. 

The results indicate that most of the UK farmers did not consider tail biting a very serious problem. UK farmers, however, perceived tail biting as a slightly more serious problem than the Finnish farmers did [13]: 64% of the UK farmers and 72% of the Finnish farmers scored the problem as not very serious (1 or 2), while 8% of the UK farmers and 3% of the Finnish farmers scored it to be a very serious problem (6 or 7). As for the Finnish study, the more tail biting the respondents reported to have occurred in the previous batch, the more serious a problem tail biting was perceived to be. However, following the same logic, it is slightly surprising that UK producers, who reported a much lower incidence of tail biting lesions (0.7%) than the Finnish producers did (2.3% [13]) still appeared to find it a more serious problem than their Finnish counterparts. It must be noted, however, that due to data restrictions in the current study, the reliability of the reported incidence of tail biting lesions is somewhat compromised.

There are several studies indicating that tail docking indeed reduces the incidence of tail lesions [37,38,39]. Thus, it was perhaps slightly surprising that in this study producers that did not dock any proportion of the tail perceived the problem of tail biting significantly less serious than producers that reared docked pigs. The problem was perceived numerically most serious by producers that reared pigs with tails docked to less than half of the original length. This might be explained by the fact that farmers who have encountered tail biting problems, or who have perceived these as more serious, consequently, have reverted to docking, or do not consider stopping tail docking to be an option due to the risk of tail biting, and might even be inclined to want the tail to be as short as possible. Docking a larger proportion of the tail has been indicated to be more efficient in reducing tail lesions than docking less [37,39]. According to the open comments, at least four respondents had tried raising intact-tailed pigs, but had then gone back to docking due to problems with biting. A second possible explanation is that producers that dock tails simply perceive tail biting as a worse problem than those that prefer intact-tailed pigs, and therefore they stick to docked pigs. Finally, this result could be explained by the producers rearing intact-tailed pigs being better at keeping tail biting to a lower level, due to differences in housing and management compared to farms rearing pigs with docked tails. This explanation is supported by the result that the reported level of tail lesion occurrence in the last batch was much lower in the set of respondents that reared long-tailed pigs, as compared to respondents that reared pigs with docked tails. 

The result in this study on how much tail biting farmers consider manageable on their farms corresponds surprisingly well with replies in the corresponding Finnish questionnaire [13]. As the tail biting level, as indicated by the respondents, was higher in the Finnish data, than in the UK data, it could be expected that the Finnish producers would also find a higher percentage of biting manageable than UK producers. This result, instead, indicates that in both countries, the expectation of most producers is indeed not that tail biting will never occur, but that it has to be kept at a manageable level. It is, however, noteworthy that both in the UK (30%) and in Finland (29%) almost a third of the respondents indicated that only a zero-level of tail biting is manageable on their farm. It is very unlikely to ever reach a situation with no tail biting risk at all within modern pig production. Therefore, it is important to communicate to producers that a zero-level of tail biting should not be an absolute prerequisite for not docking [9].

The open comments appear to confirm previous studies showing that tail biting is multifactorial, and difficult to manage. The comments also support the fact that there is a difference between farms in how big a problem they see tail biting to be. Interestingly, especially among producers that currently raise pigs with docked tails, tail docking was frequently seen as an animal welfare-promoting measure, as tail biting causes welfare problems for the pigs. Not to dock was seen as cruel to the animals. In the corresponding Finnish study [13], farmers who stated that they would *not* want to dock tails even if it was legal in Finland often motivated this by referring to docking as being cruel or inhumane. This discrepancy is possibly related to the fact that current practices are often defended [16], and that the docking practices in these two countries differ.

Regrettably, the response rate of the study was rather low. This means the results need to be considered with some care, as it is possible that, for example, producers with very high levels of tail biting did not feel motivated to enter the study. Further, as mentioned previously, there were several data restrictions regarding the indicated level of tail biting on the farm, meaning that compromises had to be made when preparing the data for analysis. The mean number of finishing pig places per farm in the current study was 2161, which is slightly above the corresponding overall figure in the UK, which is 1548, with 90% of farms having 390 to 3020 pig places in the farm [40]. The proportion of farms not docking pigs corresponds rather well with other estimates of the situation in the UK; Hunter et al. [29] reported 12.9% of the producers in their study do not dock, and in a recent paper by DeBriyne et al. [2], it was estimated that 16% of pigs in the UK are not docked.

## 5. Conclusions

The current study confirms the importance of a multifactorial approach to prevent tail biting. It also confirms earlier studies indicating that producers might rank the importance of preventive measures differently to scientists and other experts. This calls for consideration when communicating with producers, and possibly for more integration of this type of experience-based knowledge when interpreting scientific results. Producer knowledge might be based on a more holistic view than that of researchers. The study also shows that the perception of how to best prevent and intervene to avoid tail biting differs between farms of different types, and that these perceptions might be influenced by the farmers´ own experiences, which again, might differ between countries and farming systems. 

## Figures and Tables

**Table 1:** Distribution of farm size according to number of finisher places.

Farm Size	Number of Farms	Percentage of Farms
<500	18	8.8
500–999	26	12.7
1000–1999	67	32.8
2000–4999	57	27.9
>4999	14	6.9

**Table 2:** Distribution of farms using different levels of bedding.

Bedding Level	Number of Farms	Percentage
None at all	47	23.0
Less than thin layer ^1^	14	6.9
Enough for a thin layer ^2^	43	21.1
Deep-litter system	70	34.3
Other ^3^	14	6.9

^1^ Some, but bedding material doesn’t cover the whole pen when thinly spread. ^2^ Enough for bedding material to just cover the whole pen when thinly spread. ^3^ Mixed replies, such as different amount of bedding to different animals, bedding amount depends on weather.

**Table 3:** Distribution of farms with differing proportion of tail removal.

Proportion of Tail Docked	Number of Farms	Percentage
None	29	14.2
Just tip	26	12.7
1/3	52	25.5
½	36	17.6
>½	28	13.7
Mixed	12	5.9

**Table 4:** Perceived importance of the different preventive measures for tail biting given in the questionnaire. The last two columns indicate the ranking of the measures according to UK (current study) and Finnish pig producers (FI) (based on Valros et al. 2016 [13]). Measures rated within the top 10 in both countries are indicated by bolded text.

Ranking of Measures					
Measure	Sub-Category ^1^	Median (Interquartile Range) ^2^	Mean (SD) ^2^	Rank UK	Rank FI ^3^
**Water available to all pigs**	FW	7 (0)	6.6 (1.1)	1	4
Providing a stocking density which is appropriate for the pen size	HE	7 (1)	6.5 (1.2)	2	11
**Taking care of animal health**	A	7 (1)	6.0 (1.5)	3	2
**Appropriate temperature in the pen**	HE	6 (2)	5.7 (1.5)	4	9
**Reducing/eliminating draughts**	HE	6 (2)	5.7 (1.6)	5	3
**Good quality piglets (healthy, even sizes)**	A	6 (2)	5.6 (1.7)	6	6
**Maintaining air quality**	HE	6 (2)	5.6 (1.6)	7	8
**Even quality of feed (e.g., each ration has the same amount of minerals)**	FW	6 (2)	5.6 (1.7)	8	7
Avoiding mixing of animals	PB	6 (2)	5.5 (1.8)	9	14
Genetics of pigs	A	6 (3)	5.5 (1.8)	10	16
Even quantity of feed	FW	6 (3)	5.3 (1.9)	11	n.a.
Use of bedding materials (such as straw, wood-shavings/sawdust, peat)	PB	6 (4)	5.1 (2.2)	12	13
Enough space around feeder for all pigs to eat at the same time	PB	6 (4)	4.9 (2.1)	13	1
Maintaining pen hygiene/cleanliness	HE	5 (3)	4.8 (1.9)	14	20
Use of objects for manipulation (such as rope, hosepipe, wellington boots)	PB	5 (4)	4.8 (2.0)	15	17
Background of piglets (housing and management in farrowing and/or weaning unit)	A	5 (3)	4.7 (2.0)	16	12
Feeding always at the same time of day	FW	5 (4)	4.2 (2.3)	17	10
Adequate light level/lighting conditions	HE	4 (3)	4.1 (1.9)	18	19
Adjusting natural light from windows	HE	4 (3)	3.6 (1.9)	19	18
Managing noise level	HE	3 (3)	3.4 (1.9)	20	15

^1^ Measure subcategories: HE: Housing and environment; PB: Pig behavior; FW: Feed and water; A: Animals. ^2^ Scale: 1 (not important) to 7 (extremely important). ^3^ Some of the questions were worded slightly different in the two questionnaires, to match local conditions.

**Table 5:** Perceived importance of the different tail biting intervention measures given in the questionnaire.

Measure	Median (Interquartile Range) ^1^	Mean (SD) ^1^
Identifying the biter	7 (1)	6.4 (1.4)
Removing the biter from the pen	7 (1)	6.3 (1.4)
Removing the bitten pig(s) from the pen	7 (1)	6.0 (1.6)
Adding objects for manipulation (such as rope, hosepipe, wellington boots)	6 (2)	5.5 (1.8)
Adding bedding material (such as straw, wood shavings/sawdust, peat)	6 (4)	5.1 (2.1)
Reducing stocking density	5 (2)	4.8 (1.9)
Making environmental interventions (e.g., adjusting lighting levels, ventilation)	5 (3)	4.5 (1.9)
Use of anti-biting substances on the tail	4 (4)	4.2 (2.2)

^1^ Scale: 1 (not important) to 7 (extremely important).

**Table 6:** Perceived effectiveness in preventing tail biting of the different manipulable materials given in the questionnaires.

Material	Type of Material ^1^	Median (Interquartile Range) ^2^	Mean (SD) ^2^
Straw	BC	5 (4)	5.3 (1.8)
Chain	O	5 (3)	4.8 (1.7)
Ball or other object attached to a chain	O	5 (4)	4.7 (2.0)
Rope	O	4 (4)	4.2 (2.0)
Ball or other object loose on the floor	O	4 (4)	4.2 (2.0)
Cardboard or paper sacks	BC	4 (4)	4.1 (2.1)
Commercial pig toy	O	3 (4)	4.0 (1.9)
Fresh wood (such as branches, a piece of tree trunk)	O	3 (4)	4.0 (2.0)
Processed wood (such as a piece of plank/timber)	O	3 (4)	3.6 (1.9)
Hay	BC	3 (3)	3.1 (2.1)
Shredded paper	BC	3 (3)	3.1 (1.8)
Sawdust/wood shavings	BC	3 (3)	3.0 (1.8)
Peat	BC	2 (3)	2.7 (1.7)

^1^ BC: Bedding and chewable materials; O: Objects. ^2^ Scale: 1 (not effective) to 7 (extremely effective).

## Appendix A. Questions Included in the Tail Damage Questionnaire

### Appendix A.1. Background Information

1. How long have you worked as a pig producer? (estimation in years)
2. Is your farm (Please tick as appropriate):
  - A finishing farm
  - A rearing/finishing farm
  - A farrow to finish farm
  - A breeding farm
3. How many finisher places do you have on your unit?
4. Is the finishing side of your unit:
  - Indoor
  - Outdoor (pigs are born outside in fields, and stay outside in open air pens. Space is more restricted than in free range systems)
  - Free Range (pigs are born outside, remain outside, and are free to roam)
  - I don’t have finishing pigs

### Appendix A.2. Tail-Biting Prevention

5. How important are the following factors for PREVENTING tail biting on your farm?
  (note that we will later ask about how to intervene when tail biting has already started)
  Please score each factor on a scale between 1–7 where 1 = not important and 7 = extremely important
  - Housing and environment
    - Maintaining pen hygiene/cleanliness
    - Appropriate temperature in the pen
    - Maintaining air quality
    - Managing noise level
    - Reducing/eliminating draughts
    - Adjusting natural light from windows
    - Adequate light level/lighting conditions
  - Pig behaviour:
    - Providing a stocking density which is appropriate for the pen size
    - Avoiding mixing of animals
    - Enough space around feeder for all pigs to eat at the same time
    - Use of bedding materials (such as straw, wood-shavings/sawdust, peat)
    - Use of objects for manipulation (such as rope, hosepipe, wellington boots)
  - Feed and water:
    - Water available to all pigs
    - Feeding always at the same time of day
    - Even quality of feed (e.g., each ration mixed has the same amount of minerals)
    - Even quantity of feed
  - Animals:
    - Good quality piglets (healthy, even sizes)
    - Genetics of pigs
    - Background of piglets (housing & management in farrowing and/or weaning unit)
    - Taking care of animal health
6. Have you noticed any factors other than those mentioned above which contribute in preventing tail biting on your unit? (Open answers)

### Appendix A.3. Tail-Biting Prevention Tail-Biting Interventions

7. If tail biting occurs in a pen, how important do you perceive the following intervention measures on your farm? Please score each factor on a scale between 1–7 where 1 = not important and 7 = extremely important
  - Identifying the biter
  - Removing the biter from the pen
  - Removing the bitten pig(s) from the pen
  - Adding bedding material (such as straw, wood shavings/sawdust, peat)
  - Adding objects for manipulation (such as rope, hosepipe, wellington boots)
  - Reducing stocking density
  - Making environmental interventions (e.g., adjusting lighting levels, ventilation)
  - Use of anti-biting substances on the tail (Please specify)
8. Have you noticed any measures other than those mentioned above to be important on your farm when tail biting has already started? (Open answers)
9. How effective do you think the following manipulable materials and objects are in preventing tail biting? Please score each factor on a scale between 1–7 where 1 = not effective and 7 = extremely effective. If you have not used any of the below on your unit please mark as ‘I do not use/have never used’
  - Bedding and chewable materials:
    - Sawdust/wood shavings
    - Straw
    - Peat
    - Hay
    - Shredded paper
    - Cardboard or paper sacks
    - Other (please specify)
  - Objects
    - Processed wood (such as a piece of plank/timber)
    - Fresh wood (such as branches, a piece of tree trunk)
    - Commercial pig toy
    - Rope
    - Chain
    - Ball or other object attached to a chain
    - Ball or other object loose on the floor
10. Have you used other materials than those mentioned above on your farm? How efficient have you found these? (Open answers)
11. How much bedding material do you estimate you provide per pen per day?
  - None at all
  - Some, but bedding material doesn’t cover the whole pen when thinly spread
  - Enough for bedding material to just cover the whole pen when thinly spread
  - Deep-litter system
12. How many pigs do you have in a typical pen on your farm?
13. What is the ratio of stock-keepers to pigs on your farm?

### Appendix A.4. Tail Biting and Tail Docking

Tail biting is defined in this questionnaire as “a tail with clear biting marks and/or blood, or the tail is clearly shortened due to biting”.

14. How serious a problem do you perceive tail biting to be on your farm? Please assign a score between 1–7 where 1 = not a serious problem and 7 = an extremely serious problem
15. How much tail biting (% of pigs) do you perceive as manageable on your unit?
  - 0%
  - 1–2%
  - 3–4%
  - 5–7%
  - 8–10%
  - 11–15%
  - over 15%
16. If you feel comfortable providing information on your levels of tail-biting, please indicate the level of tail biting in your most recent batch. (This question is completely optional)
17. What proportion of the tail is removed on your pigs:
  - None of the tail is removed
  - Just the tip is removed
  - 1/3 of the tail is removed
  - 1/2 of the tail is removed
  - More than 1/2 of the tail is removed
  - Mixed
18. Do you have any other comments? (open answer)
